# Lying position classification based on ECG waveform and random forest during sleep in healthy people

**DOI:** 10.1186/s12938-018-0548-7

**Published:** 2018-08-30

**Authors:** Hongze Pan, Zhi Xu, Hong Yan, Yue Gao, Zhanghuang Chen, Jinzhong Song, Yu Zhang

**Affiliations:** China Astronaut Researching and Training Center, Beijing, China

**Keywords:** ECG waveform, Sleep, Lying position, Random forest, Classification

## Abstract

**Background:**

Several different lying positions, such as lying on the left side, supine, lying on the right side and prone position, existed when healthy people fell asleep. This article explored the influence of lying positions on the shape of ECG (electrocardiograph) waveform during sleep, and then lying position classification based on ECG waveform features and random forest was achieved.

**Methods:**

By means of de-noising the overnight sleep ECG data from ISRUC website dataset, as well as extracting the waveform features, we calculated a total of 30 ECG waveform features, including 2 newly proposed features, S/R and ∠QSR. The means and significant difference level of these features within different lying positions were calculated, respectively. Then 12 features were selected for three kinds of classification schemes.

**Results:**

The lying positions had comparatively less effect on time-limit features. QT interval and RR interval were significantly lower than that in supine (${\text{P}}\, \le \,0.01$). Significant differences appeared in most of the amplitude and double-direction features. When lying on the left side, the height of P wave and T wave, QRS area and T area, the QR potential difference and ∠QSR were significantly lower than those in supine (${\text{P}}\, \le \,0.01$). However, S/R was significantly greater on left than those in supine (${\text{P}}\, \le \,0.01$) and on right (${\text{P}}\, \le \,0.05$). The height of T wave and area under T wave were significantly higher in supine than those on right (${\text{P}}\, \le \,0.01$). For the subject specific classifier, a mean accuracy of 97.17% with Cohen’s kappa statistic κ of 0.91, and AUC > 0.97 were achieved. While the accuracy and κ dropped to 63.87% and 0.32, AUC > 0.66, respectively when the subject independent classifier was considered.

**Conclusions:**

When subjects were lying on the left side during sleep, due to the effect of gravity on heart, the position of heart changed, for example, turned and rotated, causing changes in the vectorcardiogram of frontal plane and horizontal plane, which lead to a change in ECG. When lying on the right side, the heart was upheld by the mediastinum, so that the degree of freedom was poor, and the ECG waveform was almost unchanged. The proposed method could be used as a technique for convenient lying position classification.

## Background

Sleep is an essential process in human life, which plays a necessary role in self-repair, self-recovery of body condition, as well as integration and consolidation of memory. It is an indispensable part of human health. About one-third of a person’s lifetime is spent during sleep. Good sleep can eliminate fatigue, restore one’s strength and energy, and ensure body functioning well. For healthy subjects during the overnight sleep, different lying positions appear such as lying on the left side, supine (lying on the back), lying on the right side, and prone (lying on the stomach). This may cause the skin to squeeze or stretch, and the distance between the electrodes to shorten or prolong. On the other hand, the heart is squeezed slightly, and chest is pressed so that breath is influenced. All these body changes will result in ECG (electrocardiograph) waveform changes.

As early as in 1997, in the course of clinical myocardial ischemia monitoring, Adams et al. had found that the side lying position frequently caused obvious ECG changes [1]. Shinar et al. found that the R-wave durations were significantly different in three lying positions, and thus successfully identified 90% of body position changes during sleep by calculating the R-wave duration of lead I, II, and III lead ECG, simultaneously [2]. Shinar further used these three leads to classify four positions, finding that the II lead ECG worked best and achieved 80% accuracy [3]. When comparing standing and supine positions of healthy subjects, Batchvarov et al. found that the RR interval of 12-lead ECG was significantly shorter in standing than that in supine [4]. Smit et al. investigated the changes of QRS waves in ECG after normal exhalation, maximum inspiration, and maximum exhalation. It was concluded that the three kinds of breath-holding conditions had little effect on the QRS complex and individual differences were large [5].

Existing studies have shown that body positions and chest changes could cause changes in ECG waveforms, but there’s no study exploring the consistent principle of such changes in ECG waveforms, systematically. It is of great importance for researchers to consider these impact in mind from lying position changing when studying the ECG waveform changes in different sleep stages. And furthermore, these changes in waveforms can be applied to non-artificial and low-intrusion lying position supervision. Consequently, in this article, we present a method of exploring the influence of lying positions on the shape of ECG waveforms during the overnight sleep in healthy subjects, and then lying position classification based on such principle and random forest is applied.

## Methods

**Fig. 1:**
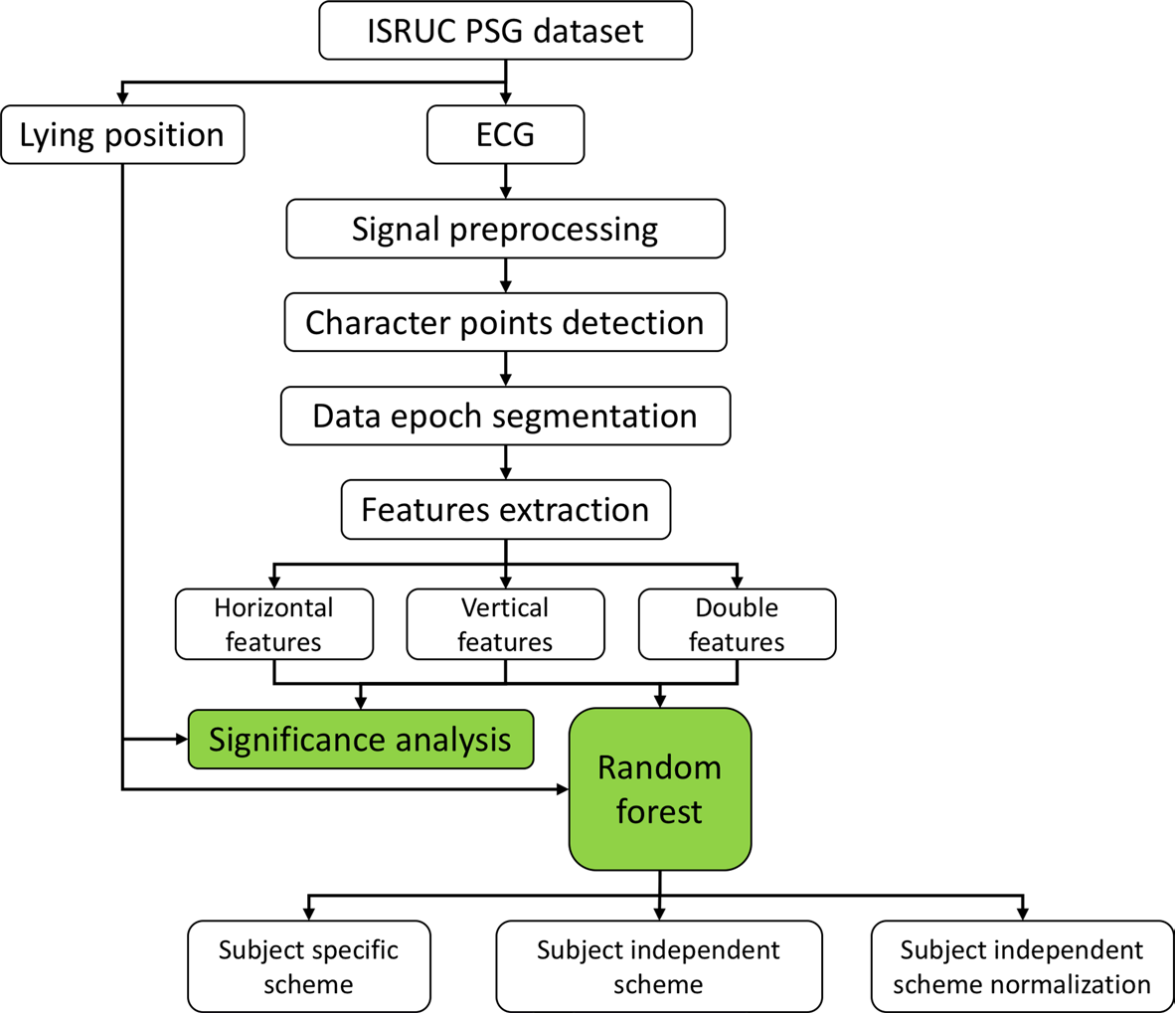
The workflow of this study

The study presented in this article can be divided into 3 parts. Data process mainly includes ECG signal preprocessing, character points detection, data epoch segmentation, features extraction with three kinds of waveform features. Then the significant differences between lying positions of waveform features are calculated. Finally lying position classification based on ECG waveform and random forest during sleep is achieved. The workflow is shown in Fig. 1.

### Dataset

The data used in this article was from the ISRUC web sleep database, which provided a variety of physiological data from 10 healthy subjects [6]. The overnight sleep data in this database was recorded by polysomnography (PSG), which lasted for about 8 h. The experiment was finished at the Sleep Medicine Center of the University of Coimbra. For each subject, the database provided a total of 19 physiological data such as electrocardiogram (ECG) and lying position. The ECG sampling rate was 200 Hz. Because the R wave peaks morphology of No. 5 subject in the database was double-peak, the determination of the R-wave peak point’s horizontal and vertical coordinates were interfered. Thus this piece of data wasn’t included in this study. For the remaining 9 participants, only a small number of subjects had prone position during the overnight sleep. Therefore, this article studied the ECG waveform changes within the left, supine and right-side lying position during the overnight sleep for 9 healthy subjects.

### Signal preprocessing

**Fig. 2:**
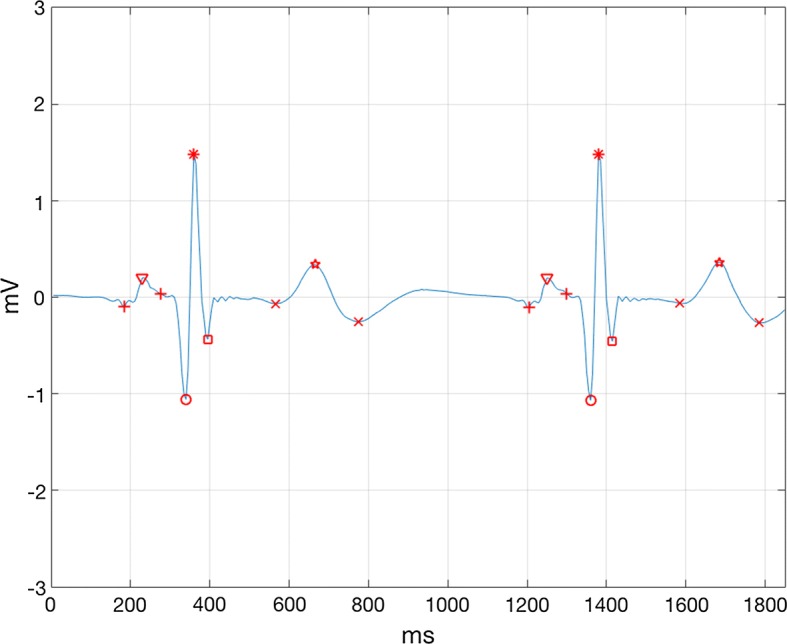
The results after signal preprocessing and character points detection. From left to right, there are P wave origin, P wave peak, P wave end, Q wave peak, R wave peak, S wave peak, T wave origin, T wave peak, T wave end. This part of ECG signal was from No.1 subject, which appeared from 5 h 40 min 11 s 505 ms to 5 h 40 min 13 s 355 ms

The ECG signal in the ISRUC database mainly contained two kinds of noises, myoelectric interference caused by muscle electrical activity with a frequency of 2 Hz–2 kHz, and baseline drift caused by human respiratory coupling. In this study, first of all, the mean filter was applied to remove the interference from AC (alternating current) in the ECG signal. Secondly, the three-layer lifting wavelet decomposition method was used to remove the high frequency myoelectric interference. Finally, the effect of baseline drift was eliminated by the function fitting method. Since this article was to explore the changes of ECG waveform features, it was necessary to acquire high accuracy point locations of P-wave, QRS-wave, and T-wave. In this study, the multi-character points detection algorithm of ECG signals based on wavelet transform, proposed by Yang et al. was used to decompose and de-noise the original signal, and the position of the QRS complex were obtained [7]. Then the area increment method, which was proposed by Song et al. was applied to locate the P wave end at the right side of P wave peak, and the T wave origin at the left side of T wave peak [8]. Finally, all the subject’s overnight ECG character points and waveforms were manually checked. After signal preprocessing and character points detection, the results are shown as follows in Fig. 2.

### Data segmentation and ECG waveform features

The ISRUC database divided the subject’s overnight sleep data into 30 s epochs. Then the sleep stage of each epoch was determined and the lying position was recorded. In this study, we excluded the time segments whose lying position duration was no longer than 1 min (two epochs), and those the ECG signal waveform disturbed during the body position changing so that the character points detection could not be performed.

The characteristics of ECG waveform morphology features and their meanings are shown in Table 1. In this study, these features are divided into three classes according to their orientation in the ECG chart, which are the time-limit features (horizontal direction features), amplitude features (vertical direction features) and double-direction features (features reflecting both time and amplitude simultaneously). The time-limit features reflect the time interval between the ECG waveforms character points on the time axis. The amplitude features reflect the height of the ECG waveforms and potential difference of points in the amplitude direction. The double-direction features mainly include area features, slope features and angle feature.

**Table 1:** The ECG waveform features explored in this study

No.	Features	Meaning	Orientation
1	QT interval	Interval between Q peak and T end	Horizontal
2	RR interval	Interval between contiguous R peak	Horizontal
3	PR inter	Interval between P begin and QRS begin	Horizontal
4	PR segment	Segment between P end and QRS begin	Horizontal
5	ST inter	Interval between QRS end and T end	Horizontal
6	ST segment	Segment between QRS end and T begin	Horizontal
7	RT slope	Slope of the line between R peak and T peak	Double
8	P wide	Wide between P begin and P end	Horizontal
9	QS wide	Wide between Q peak and S peak	Horizontal
10	T wide	Wide between T begin and T end	Horizontal
11	TP segment	Segment between T end and next P begin	Horizontal
12	P peak	Amplitude of P peak	Vertical
13	R peak	Amplitude of R peak	Vertical
14	T peak	Amplitude of T peak	Vertical
15	T area	Area under T wave	Double
16	Rp-Tp x	Wide between R peak and T peak	Horizontal
17	Rp-Tp y	The difference of amplitude between R and T	Vertical
18	Tp-Te	Wide between T peak and T end	Horizontal
19	QR	Difference of amplitude between Q and R	Vertical
20	RS	The difference of amplitude between R and S	Vertical
21	QRS area	Area under QRS wave	Double
22	S peak	Amplitude of S peak	Vertical
23	RS slope	Slope of the line between R peak and S peak	Double
24	S/R	Amplitude ratio of S and R	Vertical
25	T/R	Amplitude ratio of T and R	Vertical
26	Ta/QRSa	Area ratio of T area and QRS area	Double
27	QRSa–Ta	Area difference of T area and QRS area	Double
28	ST slope	Slope of the line between J point and T begin	Double
29	QTc	Corrected QT interval	Horizontal
30	Angle qsr	Angle of ∠QSR	Double

The calculation methods for several special waveform features are described as follows.

#### a. Waveform height features

The height of the waveform reflects the amplitude of the electrical signal. In actual ECG signal, the amplitude of the reference equipotential is not zero, and it fluctuates within a certain range. Therefore, the heights of P wave, R wave, S wave, and T wave cannot be directly represented by the vertical coordinates of waveform points. It is necessary to calculate the reference equipotential amplitude and the amplitude of each waveform with respect to the reference equipotential line. In TP segment all myocardial cells are at rest, so that there is no potential difference between them, and almost no electrical activity appears. TP segment is longer and more stable than PR segment, so TP segment was selected in this study to calculate the baseline equipotential line.

Firstly, the mean filter was selected with width 5 to smooth the TP segment. Then we selected 5 points $\left( {{\text{TP}}\left( {\text{i}} \right),\,{\text{i}}\, = \,1,\,2,\,3,\,4,\,5} \right)$ at equal intervals in the TP segment. The average amplitude of this 5 points was recorded as a stable point, which was used to represent the baseline equipotential of the corresponding ECG waveform before this TP segment. Finally, the potential difference between the P wave, R wave, S wave and T wave peaks and the stable point was calculated as the height of the corresponding waveforms. Take R wave height as an example, the waveform height formula is as follows:

1 $$\begin{aligned} R_{p} \, & = \,R\, - \,stable \\ & = \,R\, - \,\frac{1}{5}\sum\limits_{i = 1}^{5} {TP(i)} \\ \end{aligned}$$

#### b. Slope features

Slope features can reflect both time and amplitude change at the same time. The absolute value of slope features will increase with the amplitude of waveform increasing, and will decrease with the time interval increasing. Taking RT slope as an example, the formula for calculating the absolute value of the slope of the connection line between the R wave peak point and T wave peak point is as follows:

2 $$RT\_slope\, = \,\left| {\frac{R\_y\, - \,T\_y}{R\_x\, - \,T\_x}} \right|$$

#### c. Area features

**Fig. 3:**
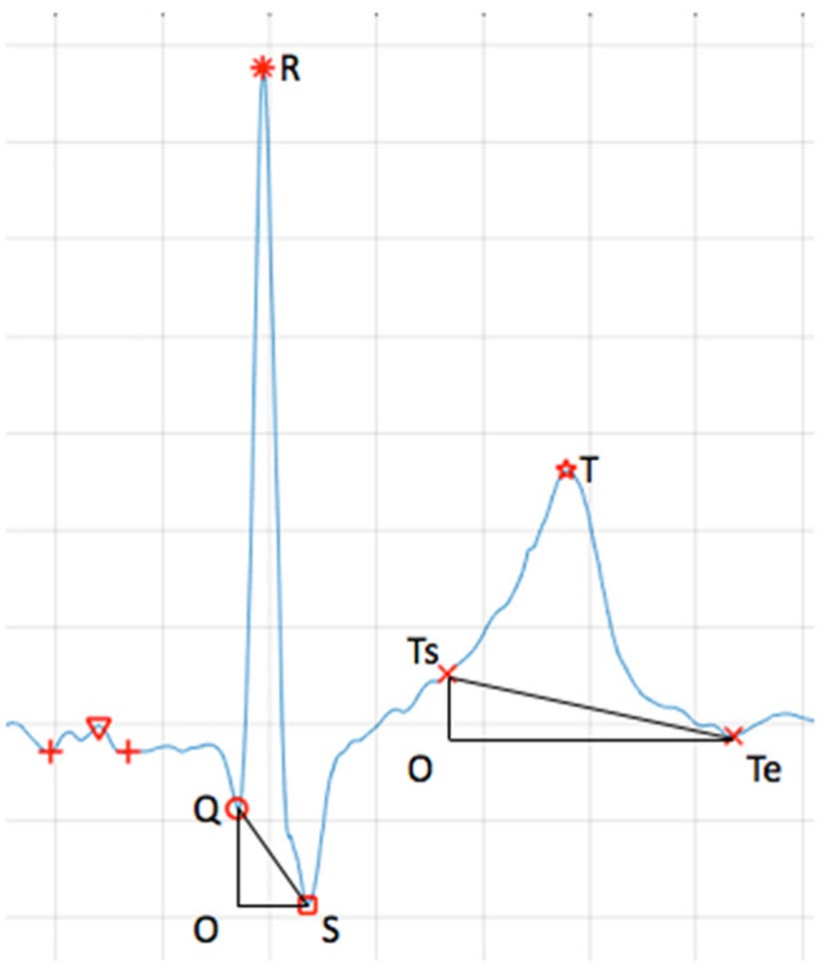
QRS complex area and T wave area calculation

In order to reduce the influence of lying position changes on the depth of Q-wave and S-wave, in this study we used the method of calculating the triangular-like area when calculating the QRS complex area. The origin of T wave might be affected by the double effect of the baseline drift and the ST segment change, resulting in different heights between the T wave start point and end point. Therefore, this method was also used when calculating the area under T wave. As shown in Fig. 3, the area of the QRS complex and the area under the T-wave should be calculated by subtracting the area of the triangle from the area obtained by summing the vertical ordinates of the ECG waveform, thereby correcting the calculation of QRS complex area and T-wave area. The formula is as follows:

3 $$\begin{aligned} QRSa & \, = \,S_{OQRS} \, - \,S_{\Delta OQS} \\ & = \,\sum\limits_{x = Q}^{S} {ecg(x)} \, - \,\frac{1}{2}\, \times \,OS\, \times \,OQ \\ \end{aligned}$$

4 $$\begin{aligned} Ta\, & = \,S_{OTsTTe} \, - \,S_{\Delta OTsTe} \\ & = \,\sum\limits_{x = Ts}^{Te} {ecg(x)} \, - \,\frac{1}{2}\, \times \,OTe\, \times \,OTs \\ \end{aligned}$$

Among them, Q represents the Q-wave peak horizontal ordinate, S represents the S-wave peak horizontal ordinate, Ts represents the beginning of the T-wave horizontal ordinate, Te represents the end of the T-wave horizontal ordinate. The meaning of other segments in the formula is shown in Fig. 3.

#### d. Corrected features

QTc (corrected QT interval) is heart-rate-corrected QT interval, that reflects the entire process of cardiac depolarization and repolarization. The calculation formula is Bazetts’s algorithm as follows:

5 $${\text{QTc}}\, = \,\frac{\text{QT}}{{\sqrt[2]{HRn}}}$$

Among the formula ${\text{HRn}}$ is the standardized heart rate. It is calculated as follows:

6 $${\text{HRn}}\, = \,\frac{60}{HR}\, = \,\frac{60}{{{{60} \mathord{\left/ {\vphantom {{60} {\frac{{\text{RRi}}}{{\text{fs}}}}}} \right. \kern-0pt} {\frac{{\text{RRi}}}{{\text{fs}}}}}}}$$

#### e. Newly proposed features

**Fig. 4:**
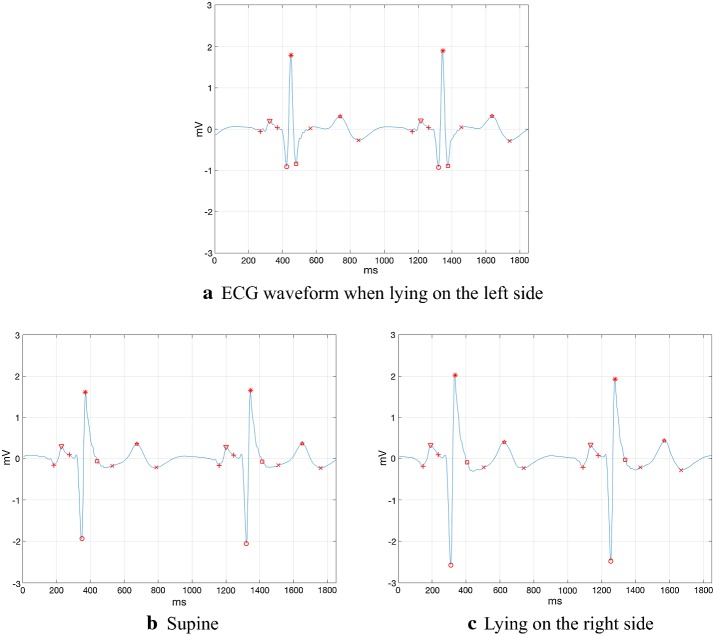
ECG waveform in 3 lying positions, all from the No.1 subject in the database. Left: from 4 h 3 min 26 s 305 ms to 4 h 3 min 28 s 155 ms. Supine: from 6 h 31 min 47 s 405 ms to 6 h 31 min 49 s 255 ms. Right: from 2 h 18 min 33 s 655 ms to 2 h 18 min 35 s 505 ms

As shown in Fig. 4, further observation on the ECG waveforms in three lying positions revealed that when lying on the left side, the S wave was lower than those in supine and lying on the right side. And the waveform amplitudes of the R waves in different lying positions were obviously different. Therefore, this study proposed two new features, namely S/R and angle ∠QSR. S/R is the ratio of S wave depth and R-wave height, which can reflect the relative depth of S waves.

7 $${{\text{S}} \mathord{\left/ {\vphantom {{\text{S}} {\text{R}}}} \right. \kern-0pt} {\text{R}}}\, = \,\frac{{{\text{S}}\, - \,stable}}{R\, - \,stable}$$

Angle ∠QSR is the angle value of the inner angle ∠QSR of the triangular QRS. Firstly, the lengths of QR, RS and QS are calculated. Then according to the cosine theorem, ∠QSR can be obtained. In this article, the unit of ∠QSR is degree, and the formula is as follows:

8 $$\angle {\text{QSR}}\, = \,{ \cos }^{ - 1} \left( {\frac{{QS^{2} \, + \,RS^{2} \, - \,{\text{QR}}^{2} }}{{2\, \times \,QS\, \times \,{\text{RS}}}}} \right)$$

### Classifier: random forest

RF (Random forest) is a novel classification method proposed by Breiman in 2001 [9]. It is a classifier that is built randomly and contains a large number of decision trees. The classification result is acquired by voting, because the output is determined by the mode of the output of each tree. Such randomness is mainly embodied in two aspects. On the one hand, a dataset of size N, which is the same as all training dataset, is selected using the bootstrapping procedure to train each decision tree. On the other hand, a subset of all features is randomly selected at each internal node. Consequently, RF can handle high-dimensional dataset (involving many features) without feature selection, and it is better at solving multiple classification problems when comparing with SVM (supporting vector machine). The decision trees are independent of each other in training procedure, so the parallel computing can be applied, which leads to fast calculation compared with ANN (artificial neural network). Besides, the structure of RF is simpler and it is easy to build, and it has strong ability to avoid over-fitting at the same time.

Because of the advantages of fast calculation, high precision, strong anti-noise ability and avoiding over-fitting when compared with other good classification method, random forest was chosen in this study. The number of trees was set as 500. After significance analysis, 12 features, including QT, RR, TP, ∠QSR, S/R, QR, P peak, R peak, T peak, T area, QRS area, T area/QRS area, were selected for classification.

When establishing each decision tree, there are two random processes to avoid over-fitting. The input data for random forest is sampled by bootstrapping procedure randomly, that is, there may be duplicate samples in the input data. Assuming N dataset, the number of input data is also N. This makes the input data of each tree not a full dataset during training, making it relatively easy to avoid over-fitting.

Then from M features, m features (m ≪ M) are randomly selected. After that, the decision tree is created by completely splitting way, so that either one leaf node of the decision tree cannot continue to split, or all the samples inside belong to the same class. Since the two random processes applied, over-fitting does not occur even without pruning. Every tree obtained by this algorithm is very weak, but they are very powerful when combined as random forest.

Each decision tree is like an expert proficient in a narrow field (because we choose m from M features to let each decision tree learn), so that there is a random forest including many experts who are proficient in different fields. When solving a new problem (new input data), they can view this from different perspective. And in the end, various experts vote to get the results. In this study, we separated the data as training data and testing data, building the RF as classifier by TreeBagger through MATLAB and the classification was achieved. We randomly selected 1–99% of the data in the database as training data, and the rest as testing data. Then the learning curves including accuracy and Cohen’ k were plotted to verifying the absence of overfitting. When the proportion of training data was more than 30%, the accuracy and Cohen’ k didn’t increase any more. But when the proportion of training data was more than 50%, the accuracy was stable and the Cohen’ k started to decrease, which meant that the overfitting existed. As we can saw in Fig. 7 in “Results” section, when the proportion was 20%, the accuracy reached a high level of 97.17% and the Cohen’ k reached an acceptable level of 0.91. Besides, less training data would lead to faster calculation. Consequently, we selected 20% of the data as training data to acquire high accuracy as well as Cohen’ k, and avoiding overfitting.

### Performance evaluation

The performance of classifier was evaluated by accuracy, Cohen’s kappa statistic κ, ROC–AUC (receiver operating characteristic curve–area under curve), Sensitivity, Specificity and F1-scores. Accuracy stands for the percentage of correctly classified epochs in the whole dataset. Statistic κ is a more effective evaluator because it takes the prior probability into account. It can be calculated as

9 $$\upkappa\, = \,\frac{{P_{A} \, - \,P_{C} }}{{P_{prio} \, - \,P_{C} }}\, = \,\frac{{\mathop \sum \nolimits_{i = 1}^{m} P_{ii} \, - \,\mathop \sum \nolimits_{i = 1}^{m} P_{i \cdot } P_{ \cdot i} }}{{P_{prio} \, - \,\mathop \sum \nolimits_{i = 1}^{m} P_{i \cdot } P_{ \cdot i} }}$$

$P_{A}$ is the proportion of correctly observed, while $P_{C}$ is the proportion of randomly expected. $P_{prio}$ is equal to 1. Such variables can be calculated by the second formula. m means the number of class. In this study m = 3. And P means the proportion of the corresponding sample to the entire. Statistic $\upkappa\, \le \,0$ means that the observed result is even worse than random expecting. And $\upkappa\, \ge \,0$ means that all sample are classified into the correct class. A higher value of κ indicates a better classification result between our classifier and the expected results.

ROC curve is a graphical plot that presents the ability of a binary classifier system. It is created by plotting the FPR (false positive rate) and TPR (true positive rate) at various threshold. Because that the classifiers in this study are ternary classifiers, after classification results are obtained, in order to draw ROC curve and calculate the AUC, Sensitivity, Specificity and F1-scores of one lying position, the other two lying positions are combined. E.g. before drawing ROC curve and calculating such several indexes of lying on the left, epochs of supine and lying on the right are combined as not-left, then the 2 × 2 confusion matrix is built.

Generally speaking, a good classifier should be associated with high values of accuracy, statistic κ and AUC.

### Classification scheme

In this study, we developed three kinds of classification scheme for different cases, including subject specific scheme, subject independent scheme without feature normalization and subject independent scheme with feature normalization. The result of ECG waveform features significance analysis between different lying positions will be presented in “Results” section. After significance analysis, 12 features, which showed strong significant difference between lying positions including QT, RR, TP, ∠QSR, S/R, QR, P peak, R peak, T peak, T area, QRS area, T area/QRS area, were selected for classification.

**Fig. 5:**
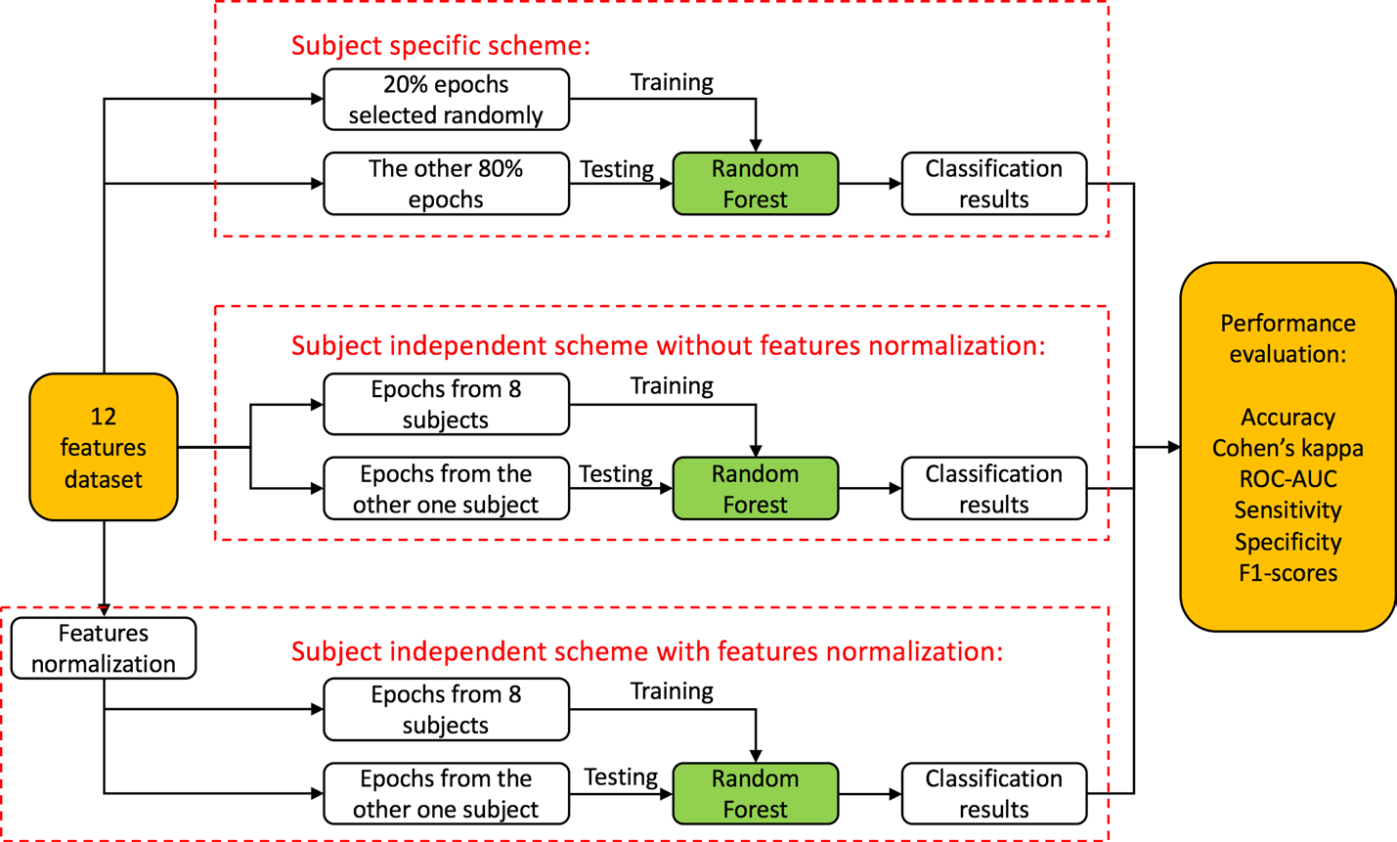
the workflow of classification method in 3 classification schemes

A total of 5114 epochs of the overnight sleep data from 9 subjects were included in this study. Due to the fact that most subjects did not have prone position, or only had several prone epochs in overnight sleep, the prone epochs were manually removed. Consequently, there are only three classes in classification including lying on the left, supine, and lying on the right. The details and workflow are shown in Fig. 5.

#### a. Subject specific scheme

For each subject, 20% epochs of three kinds of lying positions were randomly selected for training the classifier, and the rest 80% were used as testing data. The reasons 20% for training and 80% for testing are that on the one hand, the waveforms were obviously different in 3 lying positions. Strong significant difference of waveform features appeared in “Results” section. On the other hand, we were trying to train the classifier with limited data. So that when putting into application, we could build a small database for patients, extracting ECG signals for only half an hour, to train the classifier. And then clinical automatic classification with high accuracy were achieved. In order to avoid the errors caused by selecting samples randomly, the training and classification processes were repeated for 10 times with different training data. At last, the average value and standard deviation of accuracy and κ statistic were calculated.

#### b. Subject independent scheme without feature normalization

For each specific subject to be analyzed, all the records from other 8 subjects were pooled together to form the training dataset. This process repeated for 9 times. Finally, the same as the specific scheme, the average value and standard deviation of accuracy and κ statistic were calculated.

#### c. Subject independent scheme with feature normalization

However, because of the individual differences, all features need normalization before classifier training. One of the most widely used normalization method is to transform all the features scales to a new range, such as [0,1]. But when the outliers of data appear, the transformed data scale will be unsymmetrical. To solve this problem, we developed a normalization method based on quantile. The 5% and 95% quantiles of data were selected firstly and the scale of these two samples was linearly transformed to [0,1], which covers 90% of the whole samples. The rest samples were transformed with the same linear coefficients.

## Results

A total of 5114 epochs of the overnight sleep data from 9 subjects were included in this study. Table 2 shows the frequency distribution of sleep stages and lying positions for these epochs. The results part mainly includes significance analysis of features and classification performance.

**Table 2:** Sleep data epochs frequency distribution about sleep stages and postures

	Wake	REM	Light sleep	Deep sleep	Sum
Left	244	336	960	827	2367
Supine	84	52	583	108	827
Right	158	292	770	473	1693
Prone	21	49	35	122	227
Sum	507	729	2348	1530	5114

### Significance analysis of features

This study calculated the 30 waveform features of the overnight ECG sleep data from 9 healthy subjects in the database, and calculated the means and standard deviations according to the four lying positions. The calculation results and significant differences between the different lying positions are shown in Tables 3 and 4, respectively. Due to the fact that most subjects did not have prone, or only had several prone epochs in overnight sleep, the standard deviations of features in prone were not shown in Table 3. On the other hand, the waveform features significance level of only three conditions, including left–supine, left–right and right–supine positions, were calculated. The P values of ECG waveform features significant level among different lying positions are shown in Table 4.

**Table 3:** Means and standard deviations of 30 ECG waveform features in 4 lying positions

Lying positions	Left	Supine	Right	Prone
QT	442.234 ± 4.763	434.916 ± 4.907	439.099 ± 5.361	427.654
QTc	446.541 ± 11.936	448.476 ± 10.903	453.259 ± 10.506	440.679
RR	985.050 ± 15.378	946.057 ± 17.053	942.002 ± 16.838	944.844
PR inter	139.201 ± 3.113	139.811 ± 3.233	139.574 ± 2.526	164.698
PR segment	21.708 ± 3.162	24.096 ± 2.731	23.382 ± 2.766	58.389
ST inter	341.285 ± 8.573	331.296 ± 8.029	327.401 ± 8.539	352.817
ST segment	77.024 ± 10.596	71.534 ± 9.446	71.020 ± 8.098	86.649
P wide	117.493 ± 2.397	115.715 ± 2.548	116.192 ± 2.580	106.308
QS wide	65.397 ± 2.820	70.183 ± 2.684	73.107 ± 2.848	54.014
T wide	264.261 ± 5.653	259.763 ± 4.260	256.381 ± 5.765	266.168
TP segment	382.120 ± 9.830	350.049 ± 11.086	342.160 ± 9.964	330.975
P peak	0.138 ± 1.414	0.233 ± 2.303	0.222 ± 1.739	0.112
R peak	1.808 ± 11.290	2.428 ± 16.243	2.291 ± 10.299	1.184
S peak	0.655 ± 4.970	0.533 ± 5.212	0.551 ± 3.110	0.206
T peak	0.547 ± 2.812	0.776 ± 3.427	0.696 ± 3.662	0.553
QRS area	266.365 ± 108.806	389.564 ± 158.961	387.565 ± 144.974	179.905
T area	165.776 ± 59.467	228.880 ± 67.021	200.339 ± 74.594	139.677
Ta/QRSa	1.129 ± 0.365	1.153 ± 0.381	0.848 ± 0.361	1.036
QRSa–Ta	100.588 ± 81.630	160.684 ± 120.856	187.226 ± 123.533	40.228
Rp-Tp x	301.607 ± 4.836	294.935 ± 4.940	295.938 ± 5.479	296.884
Rp-Tp y	21.477 ± 10.474	28.500 ± 15.368	27.279 ± 9.478	11.814
Tp-te	116.747 ± 1.705	115.795 ± 1.878	118.661 ± 2.742	108.543
QR	3.228 ± 18.543	4.661 ± 30.424	4.431 ± 20.729	2.678
RS	2.463 ± 14.825	2.961 ± 18.830	2.841 ± 12.079	1.390
RT slope	0.361 ± 0.185	0.489 ± 0.267	0.472 ± 0.180	0.199
RS slope	5.142 ± 2.698	5.600 ± 3.576	5.106 ± 3.137	3.527
ST slope1	0.106 ± 0.097	0.116 ± 0.108	0.080 ± 0.039	− 0.028
S/R	0.365 ± 0.151	0.232 ± 0.154	0.258 ± 0.122	0.174
T/R	0.245 ± 0.177	0.242 ± 0.090	0.220 ± 0.100	0.336
Angle qsr	106.798 ± 29.477	126.956 ± 25.830	127.175 ± 24.154	127.711

In this table, the time-limit features are calculated in millisecond (ms), the amplitude features are calculated in millivolt (mV), and the angle indicator is calculated in degree. Due to the fact that most subjects did not have prone, or only had several prone epochs in overnight sleep, the standard deviations of features in prone were not shown in this table

**Table 4:** The P value of ECG waveform features significant level among different lying positions

	Left–supine	Left–right	Supine–right
QT	0.0059**	0.1341	0.2947
QTc	0.1148	0.0229*	0.1207
RR	0.0066**	0.0206*	0.4106
PR inter	0.3549	0.0881	0.9203
PR segment	0.1287	0.0222*	0.7313
ST inter	0.1023	0.1906	0.3491
ST segment	0.2737	0.3323	0.5255
P wide	0.1912	0.1905	0.2587
QS wide	0.1157	0.0975	0.3296
T wide	0.2199	0.0609	0.3164
TP segment	0.0111*	0.0182*	0.3068
P peak*	0.0028**	0.0039**	0.1516
R peak*	0.0143*	0.0235*	0.1003
S peak*	0.0360*	0.0831	0.6562
T peak*	0.0002***	0.0180*	0.0004***
QRS area*	0.0063**	0.0228*	0.2293
T area*	0.0000***	0.0814	0.0032**
T a/QRS a*	0.4441	0.0435*	0.0379*
QRSa–Ta*	0.0562	0.0369*	0.3981
Rp-Tp x	0.0191*	0.0696	0.4688
Rp-Tp y*	0.0307*	0.0316*	0.1696
Tpte	0.3207	0.3413	0.2490
QR*	0.0066**	0.0111*	0.1585
RS*	0.0418*	0.1086	0.0651
RT slope*	0.0244*	0.0205*	0.1921
RS slope*	0.2182	0.4109	0.0197*
ST slope*	0.0126*	0.4443	0.1886
S/R*	0.0014**	0.0207*	0.3539
T/R*	0.4710	0.2809	0.2560
Angleqsr*	0.0012**	0.0275*	0.4710

The first column in this table includes 30 waveform features. To facilitate the observation, the amplitude features and double-direction features are marked by *. Columns 2, 3, and 4 show the significant level of the waveform features between two lying positions. *** ${\text{P}}\, \le \,0.001$, ** ${\text{P}}\, \le \,0.01$, and * ${\text{P}}\, \le \,0.05$

### Classification performance

After significance analysis, 12 features, which showed strong significant difference between lying positions including QT, RR, TP, ∠QSR, S/R, QR, P peak, R peak, T peak, T area, QRS area, T area/QRS area, were selected for classification. Table 5 gives the confusion matrices of all individuals for subject specific scheme and subject independent scheme without or with feature quantile normalization. The numbers in Table 5 refers to the amount of epochs of target position while classified as output position.

**Table 5:** Confusion matrices based on 12 features

Output position	Target position			
	Left	Supine	Right	Sum
(a) Subject specific scheme				
Left	1868	39	7	1914
Supine	8	603	9	620
Right	14	15	1336	1365
Sum	1890	657	1352	3899
(b) Subject independent scheme without feature normalization				
Left	1492	458	247	2197
Supine	716	203	1068	1987
Right	79	166	378	623
Sum	2287	827	1693	4807
(c) Subject independent scheme with feature normalization				
Left	1757	261	334	2352
Supine	288	377	459	1124
Right	312	176	892	1380
Sum	2357	814	1685	4856

Confusion matrices based on 12 features for (a) subject specific scheme, (b) subject independent scheme without feature normalization, (c) subject independent scheme with feature normalization

**Fig. 6:**
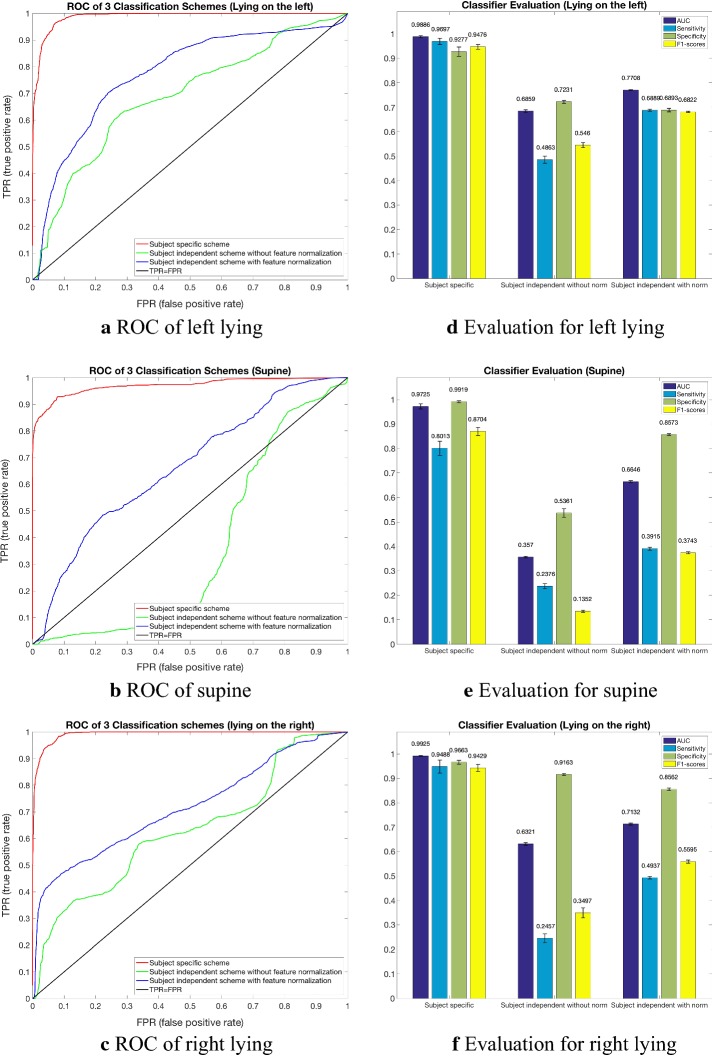
The classifier performance of three schemes. Graphs (**a**–**c**) are the ROC curves of three kinds of lying position. The red line represents subject specific scheme, green line represents subject independent scheme without features normalization and blue line represents subject independent scheme with features normalization. Bar charts (**d**–**f**) present the mean value of AUC, Sensitivity, Specificity and F1-scores of 10 experiments

Table 6 shows the classification performance based on 12 features for subject specific scheme and subject independent scheme without or with feature normalization. The process repeated 10 times, and the means and standard deviation were calculated and listed in Table 6. Figure 6 shows the classifier performance of three scheme: (a–c) show the ROC curve of 3 lying positions respectively, and (d–f) show the AUC, Sensitivity, Specificity and F1-scores of the classification result. The AUC of three lying positions in subject specific scheme reached at 0.9886 ± 0.0043, 0.9725 ± 0.0106 and 0.9925 ± 0.0019, respectively. While in subject independent scheme without features normalization 0.6859 ± 0.0050, 0.3570 ± 0.0035, 0.6321 ± 0.0055, and in subject independent scheme with features normalization 0.7708 ± 0.0017, 0.6646 ± 0.0047, 0.7132 ± 0.0040.

**Table 6:** Classification performance based on 12 features

Left	Supine	Right	Overall	κ statistic
(a) Subject specific scheme				
98.71% ± 2.03%	72.22% ± 23.41%	98.46% ± 2.34%	97.17% ± 2.74%	0.9121 ± 0.1010
(b) Subject independent scheme without feature normalization				
55.22% ± 43.25%	38.38% ± 41.36%	24.21% ± 37.28%	44.73% ± 31.61%	0.0866 ± 0.2180
(c) Subject independent scheme with feature normalization				
75.04% ± 24.10%	46.40% ± 35.61%	44.34% ± 38.14%	63.87% ± 16.32%	0.3171 ± 0.1755

Classification performance based on 12 features for (a) subject specific scheme, (b) subject independent scheme without feature normalization, (c) subject independent scheme with feature normalization

**Fig. 7:**
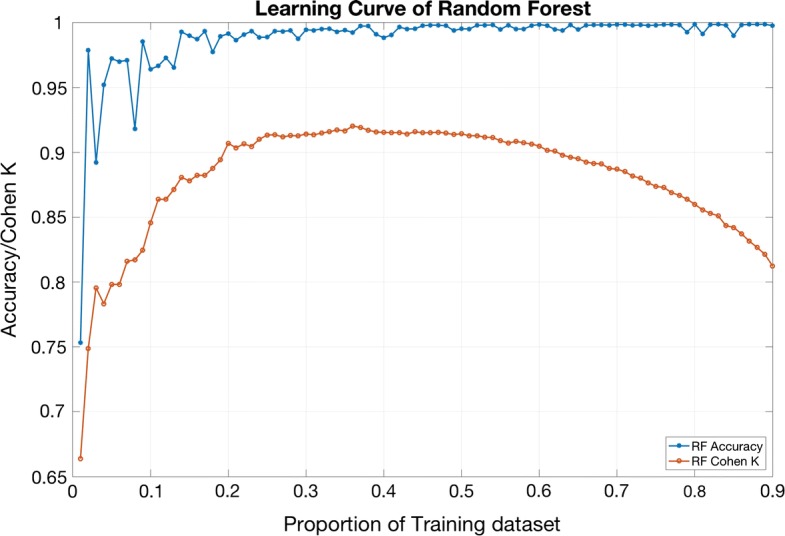
In order to verify the absence of overfitting, the learning curve are shown above. The blue line and red line represent the accuracy and Cohen’ K, respectively, of the classification result based on random forest with different proportion of training data

**Fig. 8:**
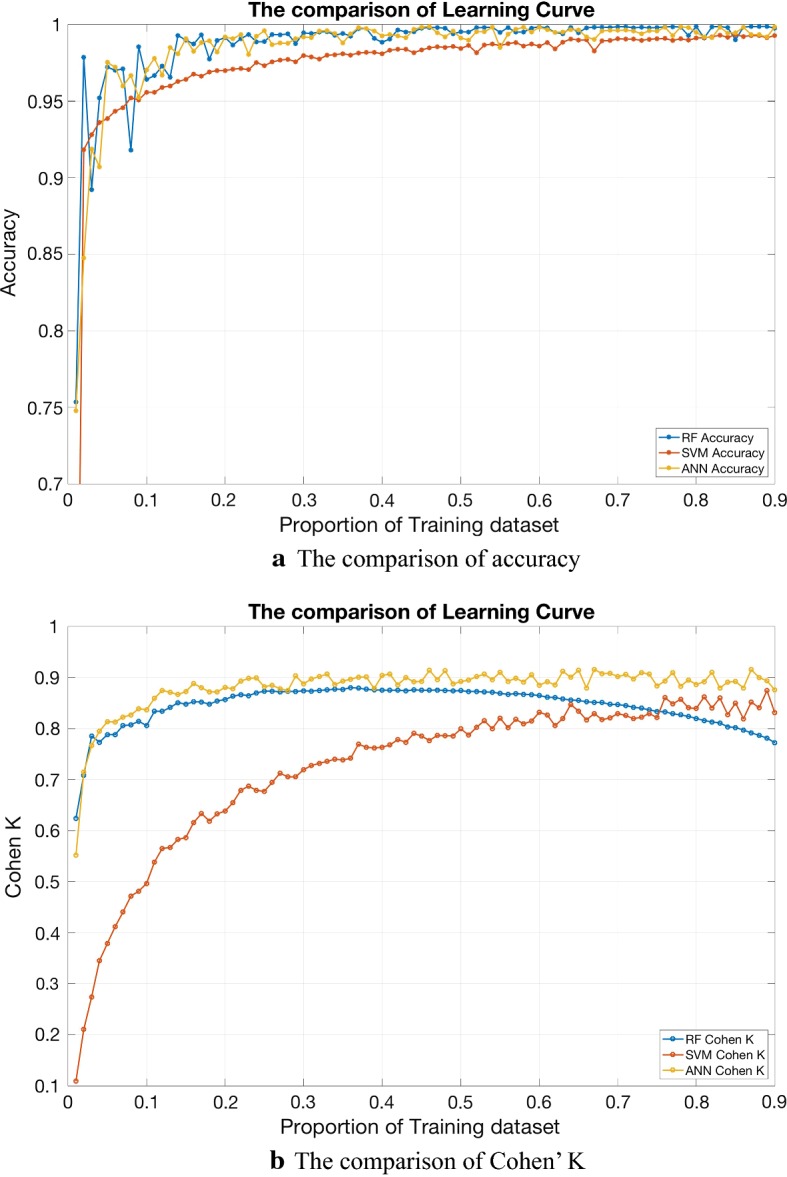
The comparison of the classification performance between RF, SVM and ANN. We can see that RM and ANN perform better than SVM, and the accuracies of RF and ANN are close. The Cohen’ k of ANN is slightly higher than RF. However, the calculation of RF is much faster. Consequently, RF performs best in general

Because the results of subject specific scheme presented in Table 6 and Fig. 6 include overall accuracy of 97.17% ± 2.74%, κ 0.9121 ± 0.1010 and AUC > 0.97 in three lying position classification), we tried to decrease the proportion of training data. The results are shown below in Table 7. In order to verify the absence of overfitting, the learning curve are shown in Fig. 7. The comparison of the classification performance between RF, SVM and ANN is shown in Fig. 8. We can see that RF and ANN perform better than SVM, and the accuracies of RF and ANN are close. The Cohen’ k of ANN is slightly higher than RF. However, according to Table 8, the calculation of RF is much faster. Consequently, RF performs best in general.

**Table 7:** Subject specific scheme with the training proportion of 0.2, 0.1 and 0.05

Left	Supine	Right	Overall	κ
(a) Subject specific scheme, the proportion of training is 0.2				
98.71% ± 2.03%	72.22% ± 23.41%	98.46% ± 2.34%	97.17% ± 2.74%	0.9121 ± 0.1010
(b) Subject specific scheme, the proportion of training is 0.1				
97.99% ± 2.74%	59.18% ± 34.50%	97.44% ± 3.02%	95.71% ± 3.38%	0.8418 ± 0.2156
(c) Subject specific scheme, the proportion of training is 0.05				
96.63% ± 6.41%	51.83% ± 37.91%	95.02% ± 4.55%	93.91% ± 4.87%	0.7902 ± 0.2546

**Table 8:** The comparison of the calculation time between RF, SVM and ANN in subject specific scheme with the training proportion of 0.2

Subject no.	RF	SVM	ANN
1	3.22291 ± 0.19684	0.04193 ± 0.00423	3.34373 ± 1.16847
2	3.02385 ± 0.12278	0.03636 ± 0.00254	3.18128 ± 0.81556
3	3.13849 ± 0.15552	0.03429 ± 0.00262	4.72749 ± 1.02038
4	2.97439 ± 0.12926	0.02707 ± 0.00118	3.10264 ± 1.28542
5	3.06737 ± 0.15559	0.03440 ± 0.00151	3.76409 ± 0.76218
6	2.83269 ± 0.13927	0.00537 ± 0.00056	2.43798 ± 0.12534
7	3.18591 ± 0.12517	0.03952 ± 0.00134	5.70489 ± 0.26425
8	3.00057 ± 0.16294	0.03204 ± 0.00114	5.50479 ± 0.32688
9	2.92328 ± 0.18162	0.03034 ± 0.00091	3.57378 ± 1.14730

The mean time and standard deviation of 10 experiments were calculated in seconds. Each value represented the mean time of all epochs lying position classification of one subject

## Discussion

### Discussions of results

The reason why we developed three kinds of schemes is that firstly we tried to establish a database which could be used for many subjects. However, because of the individual difference, the results were not acceptable. Consequently, we applied the normalization method to transform all the features scales to a new range. The results of subject independent scheme with feature normalization were much better but the accuracy was still not enough for clinical application. Finally, we developed the subject specific scheme, which was similar to building a database with the ECG data from a specific subject and then classifying the lying positions for this subject based on the database. That’s why the results were acceptable and this method could be applied in clinical monitoring.

As can be seen from Table 4, the lying positions have less influence on time-limit features, because most of the time-limit features show no significant differences between different body lying positions. Compared with supine, only QT interval, RR interval, and TP segment are significantly shorter when lying on the left side. The reason needs further exploration.

It can be seen that the influence of lying position on ECG waveforms is mainly reflected in the amplitude features and double-direction features. The amplitude features include the heights of P wave, R wave, and T wave. The relative height features include QR potential difference, RS potential difference, R peak T peak potential difference, and RT slope. Area features includes QRS complex area and T wave area. These three types of amplitude features were significantly smaller when lying on the left side than those in supine or right, or less than those in other two lying positions simultaneously. Only a few features show significant differences between supine and lying on the right side.

However, the S-wave-related waveform features are different. When lying on the left side, the depth of S wave is significantly greater than that in supine, and S/R is significantly greater than that both in supine and right. This feature reflects the decrease of R wave and the deepening of S wave in left-side lying. ∠QSR is significantly smaller in left than that in supine and right. This feature reflects the difference between the relative depth of the Q wave and S wave.

The influence of lying positions on ECG waveforms is mainly reflected in the amplitude features. Since the ECG waveform directly reflects the potential difference of the leads, and the signal is extracted from the electrodes on body surface, the body position changes will cause a change of relative position between the electrodes and heart. Thus ECG waveform morphology changed. This change can be embodied in two aspects. On the one hand, when the chest is under pressure, the distribution of body fluids changes, so that the impedance of the chest changes. Also the heart is squeezed and deformed. On the other hand, the heart is affected by gravity when lying on the side. Different parts of heart have different degree of freedom, which results in heart rotation and swing.

The significant differences of ECG waveform features in 3 lying positions could be utilized for automatic lying position classification during sleep. For three kinds of schemes, the overall classification accuracy of subject specific scheme reached 97.17%, κ statistic 0.91 and AUC > 0.97, which was almost perfect. This can be used for clinical lying position monitoring after setting up a subject specific dataset. Further study in Table 7 showed that such dataset didn’t need to be large, and the performance could be acceptable. The results of subject independent scheme without or with feature normalization were accuracy 44.73% and 63.87%, κ statistic 0.09 and 0.32, respectively. The classification accuracy of three lying positions in subject independent scheme was much better with feature normalization when compared with the results without feature normalization. On the other hand, the classification accuracy of lying on the left side was higher than those in supine and right. This can be applied for avoiding left lying in some patients with specific diseases, clinically.

The accuracy of classification results may be influenced by the ECG quality. Firstly, in order to distinguish the horizontal features (several time features were < 0.1 s), we chose the dataset with sampling rate 200 Hz. This could make sure that the time resolution was 0.005 s. Secondly, when the subjects were turning over during sleep, the signal was disturbed severely and we had to discard this epoch. But when the subjects were not changing their lying position, the signal was stable. Thirdly, we applied signal preprocessing based on wavelet transform, and it worked well. At last, the ECG signal acquisition technology is mature in recent years. As mentioned above, the ECG signal quality was good enough for this study, which could be reflected in the accuracy of character points detection.

### The structure of heart and vectorcardiogram

The bottom of heart in anatomical mainly consists of left atrium and a small part of right atrium, where the aorta and pulmonary artery cross [10]. Because of this structure, the bottom of heart in the thorax is comparatively fixed, while ventricular and the apex of heart are comparatively free. When the lying position changes or the diaphragm contracts, the heart apex will swing to a limited extent. This leads to the direction of electrocardial vector change, and so that it’s projection, ECG, changes.

**Fig. 9:**
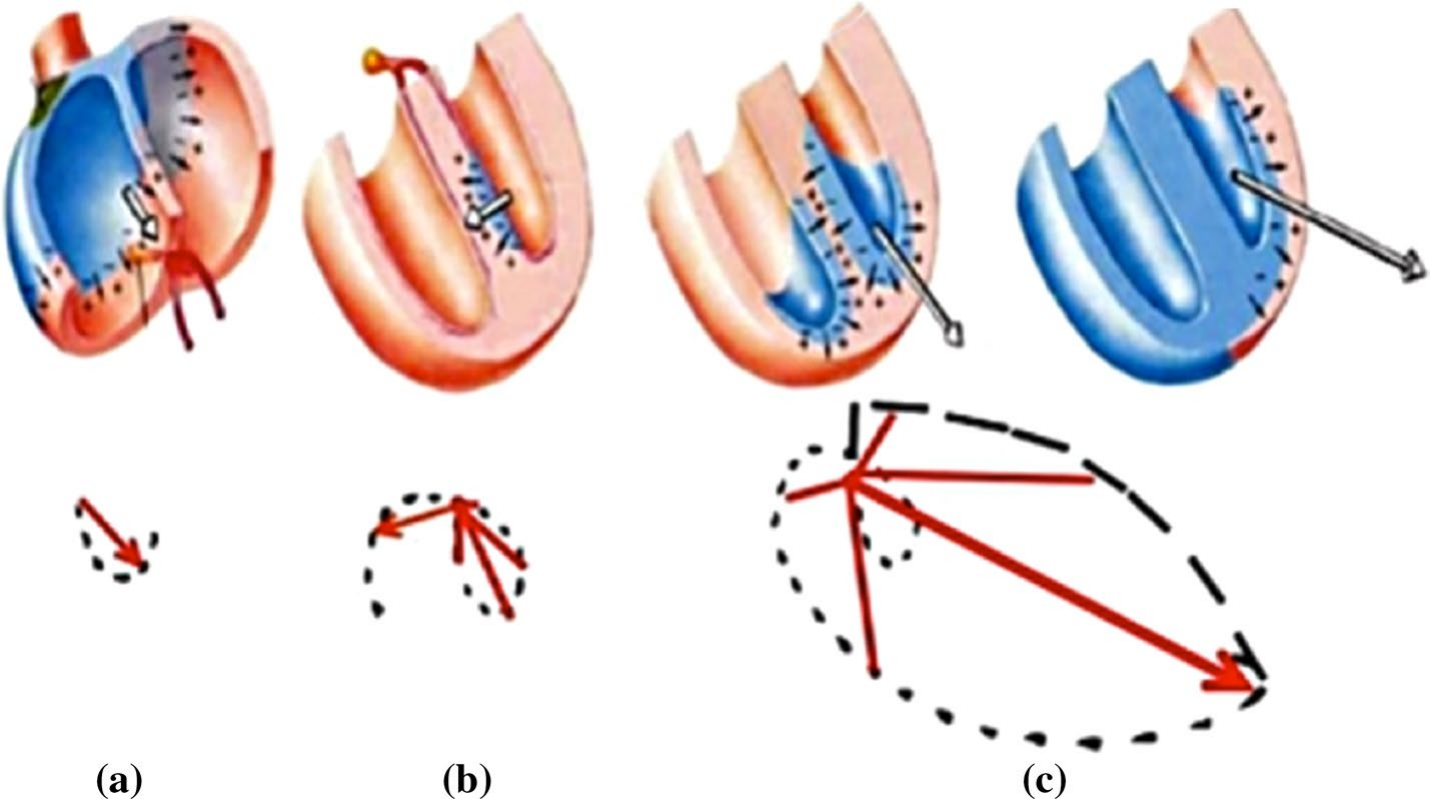
The formation of VCG (vectorcardiogram)

In a complete cardiac cycle, action potential begins from the sinoatrial node firstly, and then passes through the anterior, middle and posterior inter-nodal tract to the atrioventricular node. During this process the electrocardial vector is always from the upper right to the lower left. The process of forming the P loop is shown in Fig. 9a. Then the action potential passes through the bundle of His to the ventricle, firstly from the left bundle branch to the inter-ventricular septum, and then from the left and right bundle branches to the left and right ventricular walls, respectively. Due to the left ventricular wall being much thicker than the right, the direction of the two vectors composition is to the lower left. The formation of QRS loop is shown in Fig. 9b, c. After the action potential arrives at the apex, it travels upward along the Purkinje fiber. In this process, the direction of electrocardial vector is still to the left. Finally, after a period of time, ions reflux inside and outside the cell membrane. The formation of T loop reflects the repolarization of ventricular. A complete ECG cycle ends.

### The causes of this phenomenon

**Fig. 10:**
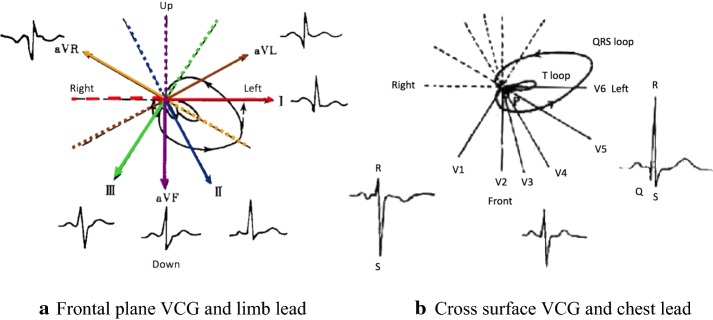
The relation between VCG and ECG

VCG intuitively reflects the direction and magnitude of the action potential vector in heart, and the ECG is actually the projection of the vector in different leads. The relationship between frontal VCG and limb lead, transverse VCG and chest lead are shown in Fig. 10a, b, respectively. The influence of lying positions on the heart can be reflected in VCG. Compared with the upright position, the position of the heart is in a relatively horizontal position when supine. As the heart rotates along the long axis (see this change in the direction from the apex to the bottom of heart, the heart rotates clockwise), the right atrium and right ventricle move left and slightly forward, and the left atrium and left ventricle are correspondingly shifted to the posterior position. The ventricular septum is almost parallel to the frontal plane instead of the side plane. View this from the frontal plane, the apex moves to the upper left and back, and the heart rotates anticlockwise along the long axis. So that there is a left-leaning tendency on the electric axis. When subjects are lying on the left side, because of the position of the bottom of heart fixed, the apex is swinging to the left, and the VCG in frontal plane is rotating anticlockwise. So that the projection lengths of P loop and T loop in lead II direction are reduced, that means, the heights of P wave and T wave in ECG waveform decrease. Reflected in the waveform features, P peak as well as T peak were significantly reduced. On the other hand, the projection length of huge part of QRS loop decreases while the tiny part increases, so the R wave of the ECG waveform becomes lower and S wave becomes deeper. Reflected in the waveform features, S/R increased while the ∠QSR decreased.

The accessible volume of heart in chest is larger when the subject is lying on the left side, because the left lung of human body is smaller than the right part and the heart is at the left side inside human chest. Therefore, the swing amplitude of heart is relatively larger. When subject is lying on the right side, the apex of the heart moves towards the mediastinum, and the heart rotates clockwise along the long axis. There shows a right-leaning tendency on the electric axis (notes: The left discussed here is the left of subject, not the left of observer). However, because the heart is upheld by the mediastinum, the range of motion is limited, so there is no obvious swing and rotation as lying on the left side. This may explain the results that waveform features rarely show significant differences between supine and lying on the right side.

### Discussions of other studies

The changes of position and shape of heart in chest have drawn the researchers’ attention. Mincholé et al. modeled the changes in the Karhunen–Loeve transform coefficients of the QRS complex and the ST–T waveform. It was found that the changes in body position can be reflected in the gradual changes of the two coefficients series. Then based on ECG, they determined the lying position changes of healthy people. The resulting probability of detection reached 94%, and the probability of false alarm was 0%, respectively. However, the false alarm rate in ischemia database was once per hour [11]. Since myocardial ischemia is widely judged by ST–T segment, the accuracy of lying position detection will decrease sharply, and the misjudgment as well as missed judgment of myocardial ischemia may be more severe if the influence of lying position on S wave morphology is not taken into consideration. Li et al. compared the heart morphology in supine and standing upright. When the subject was in supine, the heart rotated clockwise along the long axis. The heart apex moved to the left and back position. But it moved in the opposite direction when standing upright. When the subjects were standing upright, the diaphragm muscles moved down, and the heart remained vertical. At this time the electrical axis shifted to the right, the SNS (sympathetic nerve system) activity increased. But PNS (parasympathetic nerve system) activity increased in supine position [12]. Sahakian et al. studied changes in frontal QRS loop and P axis in standing upright, sitting, walking, supine, and two kinds of side lying conditions, and specialized the difference between left-side, supine and right-side lying positions, which confirmed the body positions’ influence on VCG. They found that the change of P wave is greater [13]. Most of the results are consistent with the results in this study. By means of MRI, Mase et al. presented the frontal and horizontal cross sections images of the chest. From these images, it could be seen that when lying on the left side or left-prone side, due to the effect of gravity, the heart moved down remarkably. But when lying on the right side or right-prone side, the position of heart showed no obvious difference with that in supine [14]. Such changes can also be seen in CT imaging [15, 16]. This could confirm the fact that the ECG waveform features rarely show significant differences between supine and right in this study.

Kutbay et al. study showed that the AHI (activity apnea-hypopnea index) and average minimum oxygen saturation (SOP) were significantly lower in supine than those in other lying positions, and the heart rate as well as average awakening index were higher [17]. George et al. found that lying on either side can significantly reduce OSA (obstructed sleep apnea) [18]. Garcia et al. found that the influence of body position on ECG waveform resulted in ST segment deformation. When lying on the left side, the R waves and T waves became larger and the S waves became deeper, which caused ST pattern misjudgment, and then led to false positive error or false negative error of myocardial ischemia determination [19].

Researchers have tried to classify lying positions form ECG, but most of them can only detect body position changes without lying position classification. Shinar et al. used the R wave duration (RWD) as indicator of body position changes for healthy subjects, who were asked to rotate between four body positions (back, left, prone and right). They could identify over 90% of the changes in body position. However, they couldn’t identify the exact body positions [2]. In their further study, the results showed over 90% correct identification of body position changes and up to sensitivity 79% and specificity 93% of body position classification when using any of the three leads, including leads I II and III. Lead II, which we used in this study, had the best performance for the classification of body position and correctly classified 80% of heartbeats. Classification did not improve for a combination of two leads [3]. In 2003, García et al. investigated two ECG signal processing methods for detecting body position changes. The spatial approach was based on VCG loop rotation angles and the scalar approach was based on the K–L transform coefficients. They could detected 95% of the body position changes by angle-based detector, whereas the KLT-based detector produces values of 89% [20].

The researchers also tried to classify lying position by other signals and sensors. In 2011, Zachary et al. presented a method for lying position classification using load cells placed under bed, which resulted in generalized accuracies of 0.68, 0.57, 0.69, and 0.33 for the back, right, left, and stomach positions respectively, and 0.92, 0.75, and 0.86 for the back/stomach, right, and left positions respectively [21]. The resulting accuracies, especially for left and right, were not precise enough for clinical application. In 2016, without differentiation of sitting and standing, 100% accuracy was achieved using random forest by Marcel et al. However, the signals were recorded by a gyroscope from an iPhone fixed with a belt around the torso, which was very intrusive for normal sleep. On the other hand, they couldn’t classify lying on the left or right, and the number of testing data segments were only 78 (sitting and standing were not included) [22]. In 2017, Timo et al. performed sleep position classification from a depth camera using bed aligned maps. They used Convolutional Neural Networks and achieved an accuracy of 94.0%. This approach directly recorded the body positions of patients and achieved high accuracy, but the apparatuses needed were complicated, the complexity of operations and the costs were so high that may not suitable for clinical and home nursery [23].

Studies about the influence of human lying position on ECG waveform during sleep can be widely applied in different field. First of all, changes of S wave and T wave can be used to correct the shape of ST–T segment, which can improve the determination accuracy of myocardial ischemia, and warn the sudden death early and effectively. Secondly, when studying the changes of ECG waveform and the related features in different sleep stages, the influence from lying position should be taken into consideration. Furthermore, in the process of collecting body signals and studying changes in physical conditions during sleep, if we can achieve lying positions determination based on ECG, the number of signal acquisition channels and the workload of researchers in monitoring process can be reduced. Also, patients will feel more comfortable. On the other hand, lying position monitoring can also prompt the patients to adjust their lying position during sleep, consciously. So that the frequency of respiratory disorders and sleep apnea events can be reduced. The occurrence of disease symptoms can probably be avoided and finally, sleep quality can be improved.

## Conclusion

In conclusion, this study explored the influence of lying positions on the shape of ECG waveform during sleep, and then lying position classification based on ECG waveform features and random forest was achieved. When subjects were lying on the left side during sleep, due to the effect of gravity on heart, the position of heart changed, for example, turned and rotated, causing changes in the VCG of frontal plane and horizontal plane, which lead to a change in ECG. When lying on the right side, the heart was upheld by the mediastinum, so that the degree of freedom is poor, and the ECG waveform is almost unchanged. The overall classification accuracy of subject specific scheme reached 97.17%, κ statistic 0.91 and AUC > 0.97, while the results of subject independent scheme with feature normalization were accuracy 63.87%, κ statistic 0.32 and AUC > 0.66, respectively. The proposed method could be used as a technique for convenient lying position classification.

## Abbreviations

ECG: electrocardiograph
PSG: polysomnography
AC: alternating current
RF: random forest
VCG: vectorcardiogram
MRI: magnetic resonance imaging
AHI: activity apnea-hypopnea index
OSA: obstructed sleep apnea
